# Polarization-entangled Bell state generation from an epsilon-near-zero metasurface

**DOI:** 10.1126/sciadv.ads3576

**Published:** 2025-02-21

**Authors:** Wenhe Jia, Grégoire Saerens, Ülle-Linda Talts, Helena Weigand, Robert J. Chapman, Liu Li, Rachel Grange, Yuanmu Yang

**Affiliations:** ^1^ State Key Laboratory of Precision Measurement Technology and Instruments, Department of Precision Instrument, Tsinghua University, Beijing 100084, China.; ^2^ Optical Nanomaterial Group, Institute for Quantum Electronics, Department of Physics, ETH Zurich, Zurich 8093, Switzerland.

## Abstract

Pairs of polarization-entangled photons are important for diverse quantum technologies, such as quantum communication, computation, and imaging. However, generating complex polarization-entangled states has long been constrained by the available nonlinear susceptibility tensor of natural materials, necessitating cumbersome setups for additional coherent superposition or postselection. In this study, we experimentally demonstrate the generation of pairs of polarization-entangled photons using a plasmonic metasurface strongly coupled to an epsilon-near-zero (ENZ) material. By engineering a resonance at the pump wavelength and leveraging the field enhancement provided by the ENZ effect, the photon pair generation efficiency of the 68-nanometer-thick metasurface is substantially boosted compared to that of an unpatterned indium tin oxide film. More notably, the ENZ metasurface platform facilitates versatile manipulation of the system’s anisotropic second-order nonlinear susceptibility tensor, enabling direct control over the polarization states of the photon pairs and generating a polarization-entangled Bell state without additional components. This approach enables simultaneous photon pair generation and quantum state engineering in a compact platform.

## Teaser

Pairs of polarization-entangled photons are realized using a metasurface strongly coupled to an epsilon-near-zero material.

## INTRODUCTION

Pairs of entangled photons are important for various modern quantum technologies, such as quantum key distribution (*1*–*3*), computation (*4*–*6*), imaging (*7*, *8*), and sensing (*9*, *10*). They can be generated via spontaneous parametric down-conversion (SPDC) (*11*, *12*) or spontaneous four-wave mixing (*13*, *14*) in either bulk crystals or integrated photonic platforms. Nevertheless, these approaches require stringent adherence to phase-matching conditions, which involve birefringence engineering, periodic poling of the crystal, or temperature regulation (*15*). Furthermore, the nonlinear susceptibility tensor of dielectric nonlinear materials, being an intrinsic material property, offers limited versatility for quantum state engineering. For example, the generation of pairs of polarization-entangled photons demands elaborate manipulation of the nonlinear crystals (*15*) or the integration with supplementary beam-splitting devices (*16*) or interferometers (*17*). This underscores the imperative for a more compact and adaptable platform that can generate and manipulate pairs of entangled photons, particularly for quantum applications in challenging environments where size and weight constraints are paramount.

Metasurfaces, which consist of artificially engineered subwavelength structures, have garnered considerable interest for applications in nonlinear optics (*18*–*21*). They can mitigate the stringent phase-matching conditions of bulk crystals and support resonances at both pump and emission wavelengths, thereby substantially enhancing the efficiency of various nonlinear processes at subwavelength scales. Recently, the evolution of nonlinear metasurface technology has ventured into the quantum realm (*22*, *23*). One advantage of using an ultrathin structure for the generation of pairs of entangled photons is that it can facilitate the SPDC process across a much wider frequency and angular spectrum compared to a bulk crystal (*24*–*27*). Furthermore, metasurfaces have the ability to manipulate light fields in multiple dimensions, which has led to the creation of photon pairs that are spectrally (*28*) or spatially (*29*–*31*) entangled, offering a high degree of versatility. In the latest advancements, a key focus has been the direct generation of pairs of polarization-entangled photons using metasurfaces. Polarization is a widely used information encoding channel in quantum communication due to its simplicity and robustness (*32*), especially in free-space systems (*33*). Despite several recent theoretical proposals (*34*, *35*), an experimental demonstration of generating a polarization-entangled Bell state with a resonance-enhanced metasurface platform has yet to be achieved, marking an important frontier between meta-optics and quantum nonlinear optics.

Nonlinear dielectric metasurfaces are typically composed of materials with complicated anisotropic nonlinear susceptibility tensors. On the other hand, the anisotropic nonlinear susceptibility tensors of plasmonic metasurfaces can be flexibly engineered by simply varying the geometry of the composing meta-atoms (*18*, *36*–*38*), thus holding great potential to manipulate the polarization states of the generated photon pairs. Nonetheless, although plasmonic metasurfaces have been widely applied for manipulating various nonlinear processes, the experimental demonstration of photon pair generation from plasmonic metasurfaces has not yet been achieved and is only theoretically proposed (*39*, *40*). The smaller mode volume and higher ohmic losses of plasmonic metasurfaces compared to their dielectric counterparts may result in a lower nonlinear conversion efficiency (*36*), which impedes their use in the SPDC process.

One solution to enhance the nonlinearity of plasmonic metasurfaces is through their strong coupling with an epsilon-near-zero (ENZ) material. In recent years, ENZ materials, characterized by a permittivity whose real part approaches zero (*41*), have been widely studied in nonlinear optics (*42*–*44*). The reduced group velocity of light at ENZ wavelengths results in substantial electric field enhancement (*45*), which has been effectively harnessed to amplify various nonlinear frequency conversion processes (*46*–*48*). Furthermore, it has been shown that coupling ENZ materials with plasmonic metasurfaces can substantially enhance the nonlinear response of the systems (*49*–*51*).

**Fig. 1.**
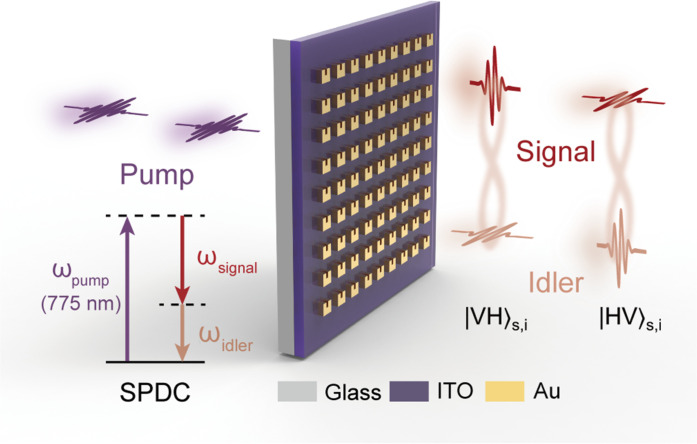
Schematic illustration of the generation of pairs of entangled photons from an ENZ metasurface via the SPDC process. By strongly coupling an ENZ material, ITO thin film, with an array of gold (Au) SRRs and engineering a resonance near the pump wavelength, the photon pair generation efficiency can be substantially enhanced. Moreover, by engineering the anisotropic second-order nonlinear susceptibility tensor of the ENZ metasurface, a polarization-entangled Bell state can be generated (V for vertical and H for horizontal).

Here, we report on the experimental generation of a polarization-entangled Bell state from a 68-nm-thick ENZ metasurface. The metasurface is composed of an array of gold (Au) split-ring resonators (SRRs) that are situated on an indium tin oxide (ITO) thin film, as depicted in Fig. 1. By leveraging strong coupling to the ENZ material and precisely engineering a resonance at the pump wavelength, the photon pair generation efficiency is substantially boosted compared to that of an unpatterned ITO film. Furthermore, we achieve control over the polarization states of the photon pairs by engineering the anisotropic second-order nonlinear susceptibility tensor of the ENZ metasurface. Notably, a polarization-entangled Bell state is directly generated from the ENZ metasurface, without requiring any additional optical components. The fidelity of this entangled state is measured to be 0.91, indicating a high degree of entanglement. This work marks a considerable advancement toward versatile polarization quantum state generation and manipulation at the nanoscale.

## RESULTS

### ENZ metasurface design and characterization

The ENZ metasurface is designed with a commercially available 23-nm-thick ITO thin film on a float glass substrate (PGO GmbH). The permittivity of the ITO thin film is measured via spectroscopic ellipsometry, revealing the real part of its permittivity crossing zero at 1420 nm (see text S1 in the Supplementary Materials for details about the linear characteristics of the ITO film).

**Fig. 2.**
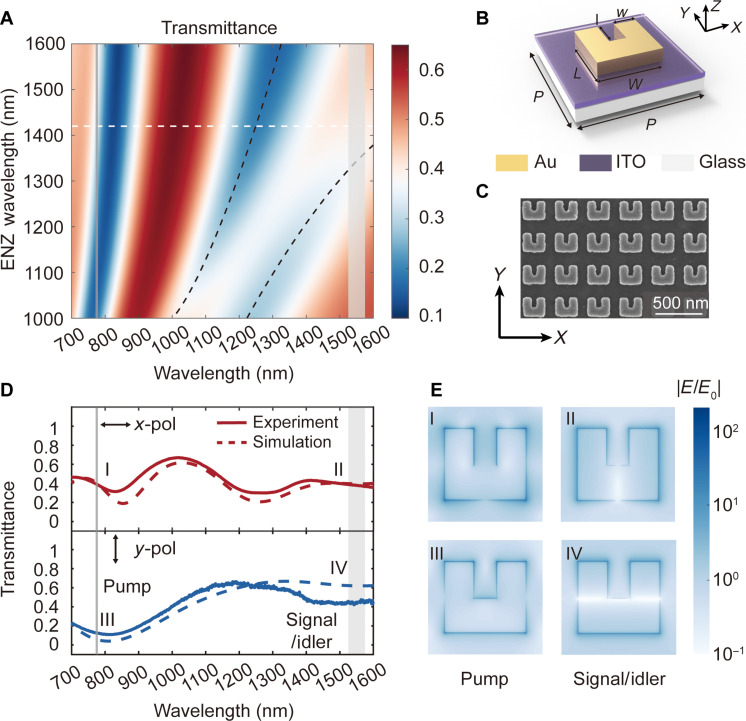
ENZ metasurface design and characterization. (**A**) Simulated linear transmittance spectra of the metasurface under *x*-polarized incident light as a function of the ITO film’s ENZ wavelength. The white dashed line indicates the measured ENZ wavelength of the ITO film, and the black dashed lines denote the anticrossing-shaped resonances. The gray solid line and band mark the pump wavelength (775 nm) and signal/idler wavelength (near 1550 nm), respectively, in the following SPDC measurements. (**B**) Schematic of a gold SRR coupled to an ITO film. The geometric parameters are detailed as follows: *P* = 350 nm, *W* = 248 nm, *L* = 217 nm, *w* = 89 nm, and *l* = 112 nm. The ITO layer is 23-nm-thick, and the gold layer is 40-nm-thick. A 5-nm-thick titanium layer is used as the adhesion layer. (**C**) SEM image of the fabricated ENZ metasurface. (**D**) Measured (solid) and simulated (dashed) transmittance spectra of the ENZ metasurface for *x*-polarized (top panel) and *y*-polarized (bottom panel) light, respectively. The gray solid line and band mark the pump wavelength (775 nm) and signal/idler wavelength (near 1550 nm), respectively, in the following SPDC measurements. (**E**) Electric field amplitude distributions at the gold-ITO interface along the *x*-*y* plane. Panels I to IV correspond to polarization states and wavelengths shown in (D).

The geometric parameters of the SRRs are meticulously varied to identify configurations that support a magnetic dipole resonance near the ENZ wavelength of the ITO film. This design results in a strongly coupled system that creates a resonance close to both the signal and idler wavelengths around 1550 nm, which is anticipated to substantially amplify the nonlinear response of the plasmonic metasurfaces. The simulated transmission spectra, as a function of the ITO film’s ENZ wavelength, exhibit an anticrossing shape of resonances near 1200 and 1600 nm (Fig. 2A), indicative of a strong coupling effect between the SRRs and the ENZ film (see text S2 in the Supplementary Materials for details about the analysis of the strong coupling effect in the ENZ metasurface). Meanwhile, an electric dipole resonance is supported at the pump wavelength of 775 nm, facilitating a resonant enhancement in photon pair generation.

Compared to the dielectric metasurfaces, the resonances and the corresponding field enhancements in a plasmonic metasurface tend to be relatively broadband, making them more forgiving in terms of fabrication tolerances. The geometric parameters of the SRRs are detailed in Fig. 2B. The nanostructure array, measuring 200 by 200 μm^2^, is fabricated through a streamlined process involving electron beam lithography, electron beam evaporation, and lift-off, with a corresponding scanning electron microscope (SEM) image presented in Fig. 2C (see Materials and Methods for details about the metasurface fabrication).

The measured polarization-dependent transmittance spectra of the ENZ metasurface are depicted in Fig. 2D. The experimental data align well with the simulations, revealing transmittance minima close to both the pump and signal/idler wavelengths. Given that gold (*52*) and ITO (*53*) have surface second-order nonlinearity, the origin of the photon pair generation is attributed to the gold-ITO interface. To gauge the field confinement and the associated SPDC enhancement, we simulated the electric field amplitude distribution at this interface. As illustrated in Fig. 2E, the electric field amplitude is substantially enhanced by over two orders of magnitude due to the strong coupling effect, a phenomenon that can be harnessed to accelerate the photon pair generation process.

### Photon pair generation measurements

**Fig. 3.**
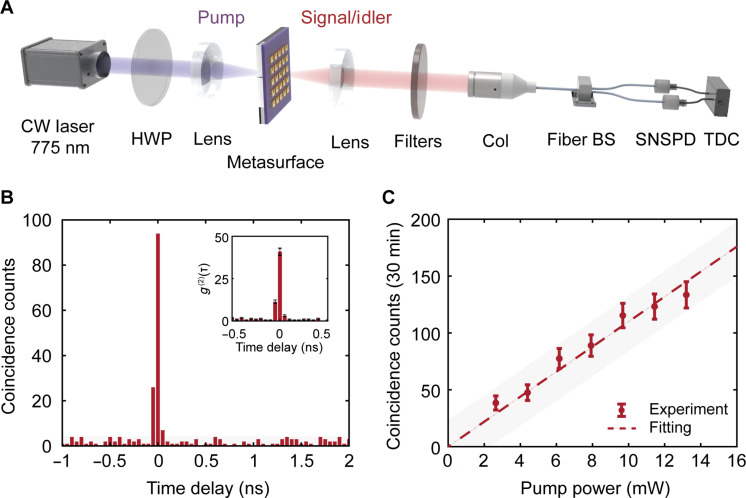
Photon pair generation and characterization. (**A**) Schematic illustration of the HBT setup. HWP, half-wave plate; Col, fiber collimator; BS, beam splitter; SNSPD, superconducting nanowire single-photon detector; TDC, time-to-digital converter. (**B**) Measured coincidence histogram from the ENZ metasurface. Inset: second-order correlation function *g*^(2)^(τ) showing a coincidences-to-accidentals ratio (CAR) of 40. (**C**) Measured coincidence counts from the ENZ metasurface (dots) and the corresponding linear fitting curve (line) as a function of the pump power.

To characterize the photon pairs generated from the ENZ metasurface, we construct a Hanbury-Brown-Twiss (HBT) experimental setup, as depicted in Fig. 3A and detailed in our previous studies (*54*, *55*). A continuous-wave (CW) laser, operating at a wavelength of 775 nm, is directed onto the ENZ metasurface through a high–numerical aperture (NA) lens (NA = 0.5). The focused laser spot has a full width at half maximum (FWHM) diameter of ~5 μm. The resulting photon pairs are collected by an identical lens, spectrally separated from the pump laser light using a combination of two long-pass filters and a band-pass filter, and then coupled into a single-mode fiber. The photon pairs are subsequently divided by a 50:50 fiber beam splitter and detected by two independent superconducting nanowire single-photon detectors (SNSPDs). The detection events are recorded using a time-to-digital converter (TDC), which compiles a coincidence histogram from the signals from both detectors (see Materials and Methods for details about the experimental setup).

Figure 3B illustrates the measured coincidence histogram from the ENZ metasurface, obtained with a pump power of 3 mW and an integration time of 2 hours. A distinct peak at zero time delay is observed, indicative of the generation of pairs of entangled photons. To substantiate the non-classical nature of the photon pairs, we calculate the second-order correlation function *g*^(2)^(τ), as shown in the inset of Fig. 3B. The corresponding coincidences-to-accidentals ratio [CAR = *g*^(2)^(0) − 1] is 40, which substantially exceeds the classical limit of 2. The measured photon pair emission rate, normalized to the pump power, is 5.4 × 10^−3^ Hz/mW. The comparison with respect to other polarization-entangled photon sources is detailed in text S3 of the Supplementary Materials. Moreover, we experimentally investigate the spectral characteristics of the generated photon pairs, showing that the generation rate remains approximately constant over a 134-nm range (see text S4 in the Supplementary Materials for details). The emission bandwidth of the ENZ metasurface is considerably broader than that of nonlinear bulk crystals, benefiting from the lack of phase-matching constraints (*24*, *27*).

Further exploration into the photon pair generation mechanism is conducted by measuring the power dependence of the coincident counts, as shown in Fig. 3C. The coincident counts demonstrate a linear dependence on the pump power, and *g*^(2)^(0) is observed to be inversely proportional to the pump power, consistent with the scaling behavior of the SPDC process [see text S5 in the Supplementary Materials for details about *g*^(2)^(0) characterization] (*56*). No coincidence peak is detected from the bare ITO film, thereby confirming that the photon pairs are indeed enhanced by the ENZ metasurface (see text S6 in the Supplementary Materials for details about SPDC measurement of the ITO film).

### Engineered anisotropic χ^(2)^ tensor of the ENZ metasurface

**Fig. 4.**
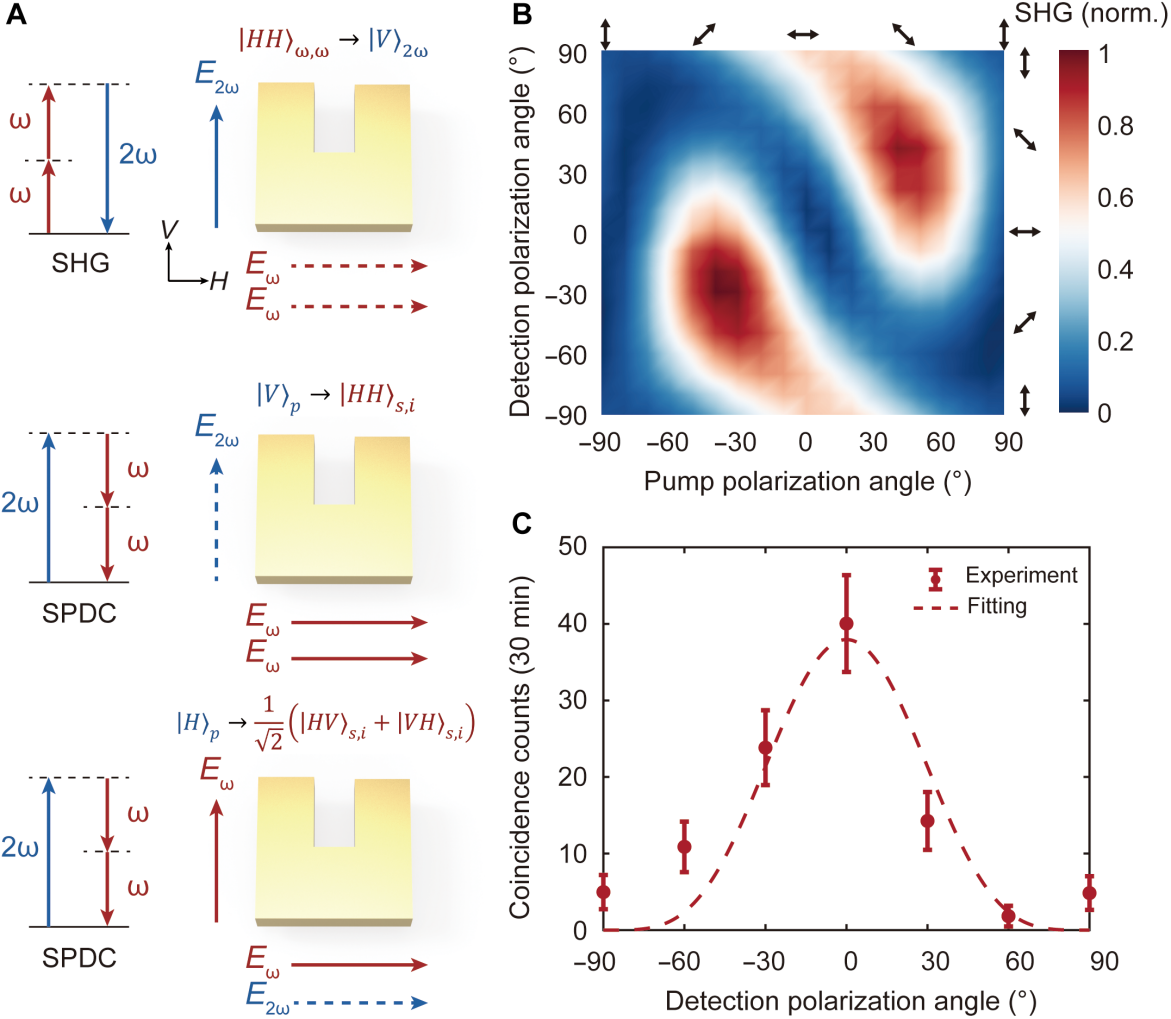
Engineered anisotropic χ^(2)^ tensor of the ENZ metasurface. (**A**) Schematic illustration of the polarization characteristics of the SHG process (top panel) and the SPDC processes with different pump configurations (middle and bottom panels). (**B**) Measured SHG intensity from the ENZ metasurface as a function of the pump and the detection polarization angles. Both the pump and detection polarization states are linearly polarized, as indicated by the arrows. (**C**) Measured coincidence counts from the ENZ metasurface under *V*-polarized pumping (dots) and the fitting curve (line) as a function of the detection polarization angle. The coincidence counts fit with the function *N* ∝ cos^4^θ, where θ is the transmissive angle of the polarizer with respect to the *H*-axis. At θ = 0°, detection is performed with *H*-polarization, whereas at θ = ±90°, detection is performed with *V*-polarization.

The SRR with broken structural symmetry (*57*) provides an opportunity to engineer the anisotropic second-order nonlinear susceptibility tensor χ^(2)^ of the ENZ metasurface. Both gold (*52*) and ITO (*46*, *53*) exhibit only a surface χ^(2)^ and have an identical tensor form. The effective second-order nonlinearity tensor of the hybrid ENZ metasurface originates from the geometric symmetry breaking (*58*). We first analyze the second-order nonlinear response of the ENZ metasurface using the reverse process of SPDC, second-harmonic generation (SHG). When exciting the SRR with *x*-polarized light, the magnetic dipole resonance can induce a continuous nonlinear current around the SRR, resulting in the generation of coherent second-order nonlinear polarization along its two bars (*57*, *58*). This process facilitates the orthogonally polarized type-1 SHG (*59*), as illustrated in the top panel of Fig. 4A. On the other hand, when exciting the SRR with *y*-polarized light, the nonlinear polarization induced from two bars of SRR interferes destructively in the far field, preventing the generation of the second-harmonic wave (*57*, *58*). Therefore, one can infer that the effective χ^(2)^ tensor, $\chi_{\mathrm{xxx}}^{\left(2\right)}=0$, $\chi_{\mathrm{yyy}}^{\left(2\right)}=0$, $\chi_{\mathrm{xyy}}^{\left(2\right)}=0$, and $\chi_{\mathrm{yxx}}^{\left(2\right)}\neq 0$. Moreover, on the basis of the Kleinman symmetry condition (*50*), one can further derive other elements in the χ^(2)^ tensor as $\chi_{\mathrm{xyy}}^{\left(2\right)}=\chi_{\mathrm{yyx}}^{\left(2\right)}=\chi_{\mathrm{yxy}}^{\left(2\right)}=0$ and $\chi_{\mathrm{yxx}}^{\left(2\right)}=\chi_{\mathrm{xxy}}^{\left(2\right)}=\chi_{\mathrm{xyx}}^{\left(2\right)}\neq 0$. The experimentally measured SHG intensity as a function of the pump and detection polarization angles, as shown in Fig. 4B, aligns well with the theoretical prediction (see Materials and Methods for details about the experimental setup and text S7 in the Supplementary Materials for details about the theoretical SHG polarization dependence).

The quantum-classical correspondence links the SPDC with its inverse SHG process, indicating that the polarization response of the SPDC process can also be estimated using the effective χ^(2)^ tensor (*60*). For instance, when pumped with *y*-polarized light, both the signal and idler photons are expected to be *x*-polarized from the only nonvanishing χ^(2)^ element $\chi_{\mathrm{yxx}}^{\left(2\right)}$, following the type-1 SPDC process, as schematically depicted in the middle panel of Fig. 4B, which can be represented as ${|V\rangle }_{p}\overset{\chi_{\mathrm{yxx}}^{\left(2\right)}}{\to }{|\mathrm{HH}\rangle }_{s,i}$. Such behavior is verified by measuring the coincident counts as a function of the detection polarization angle when pumped with *y*-polarized light, as shown in Fig. 4C (see text S8 in the Supplementary Materials for details about the theoretical SPDC polarization dependence).

On the other hand, if the ENZ metasurface is pumped with *x*-polarized light, the generated signal and idler photons are expected to exhibit orthogonal linear polarizations, as illustrated in the bottom panel of Fig. 4A. This type-2 SPDC process occurs through two pathways: ${|H\rangle }_{p}\overset{\chi_{\mathrm{xxy}}^{\left(2\right)}}{\to }{|\mathrm{HV}\rangle }_{s,i}$ and ${|H\rangle }_{p}\overset{\chi_{\mathrm{xyx}}^{\left(2\right)}}{\to }{|\mathrm{VH}\rangle }_{s,i}$, each with equal probabilities. The resulting state is a coherent superposition of states from these two pathways, represented as ${|H\rangle }_{p}\to \frac{1}{\sqrt{2}}\left({|\mathrm{HV}\rangle }_{s,i}+{|\mathrm{VH}\rangle }_{s,i}\right)$, indicating the generation of a maximally entangled quantum state, namely, Bell state.

### Polarization-entangled Bell state generation measurement

**Fig. 5.**
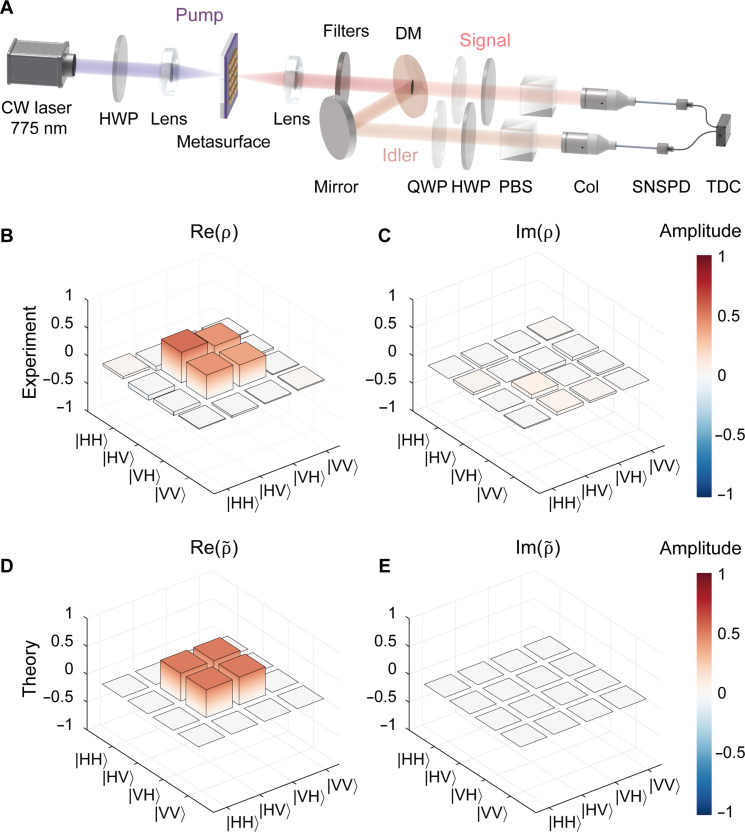
Polarization-entangled Bell state generation from the ENZ metasurface. (**A**) Schematic illustration of the quantum state tomography setup. DM, dichroic mirror; QWP, quarter-wave plate; PBS, polarizing beam splitter. (**B** and **C**) Real (B) and imaginary (C) parts of the reconstructed density matrix ρ of the polarization state of the photon pairs. (**D** and **E**) Real (D) and imaginary (E) parts of the theoretical density matrix $\tilde{\rho }$ of the Bell state $|\psi \rangle =\frac{1}{\sqrt{2}}\left({|\mathrm{HV}\rangle }_{s,i}+{|\mathrm{VH}\rangle }_{s,i}\right)$.

To completely determine the polarization states of the generated photon pairs and confirm the generation of a polarization-entangled Bell state under *x*-polarized pump, we perform a quantum state tomography measurement, with the setup schematically shown in Fig. 5A. A broadband dichroic mirror with a cutoff wavelength of 1550 nm is used to deterministically separate the signal and idler photons. Two sets of quarter-wave plates, half-wave plates (HWPs), and polarizing beam splitters are used to fully characterize the polarization states of the signal and idler photons individually. We choose 16 sets of basic polarization states for the measurement, which allows the reconstruction of the density matrix for the quantum state of the photon pairs (see text S9 in the Supplementary Materials for details about the measurement) (*32*). The real and imaginary parts of the reconstructed density matrix are shown in Fig. 5 (B and C), respectively. Compared with the theoretical density matrix for the Bell state $|\psi \rangle =\frac{1}{\sqrt{2}}\left({|\mathrm{HV}\rangle }_{s,i}+{|\mathrm{VH}\rangle }_{s,i}\right)$ (shown in Fig. 5, D and E), the calculated fidelity is 0.91, indicating a high degree of entanglement (see text S9 in the Supplementary Materials for details about the fidelity calculation method and its pump polarization dependence).

## DISCUSSION

To summarize, we experimentally demonstrate photon pair generation via the SPDC process from a 68-nm-thick plasmonic metasurface strongly coupled to an ENZ material. By tailoring a resonance at the pump wavelength and leveraging the field enhancement induced by the ENZ effect, the generation efficiency of the plasmonic metasurface is greatly boosted compared to that of an unpatterned ITO film. The CAR of the photon pairs is 40, substantially exceeding the limit of classical light radiation. Moreover, by engineering the system’s anisotropic nonlinear susceptibility tensor, we manipulate the polarization state of the generated photon pairs. A polarization-entangled Bell state is generated using the ENZ metasurface, showing a measured fidelity of 0.91.

The metasurface platform, with its distinctive capability to have a tailorable anisotropic nonlinear susceptibility tensor, opens an avenue for achieving miniaturized sources of pairs of entangled photons with complex quantum states unattainable with conventional approaches. Looking ahead, we could use resonances with higher quality factors such as quasi-bound states in the continuum resonances (*61*) or surface lattice resonances (*62*) to achieve greater field enhancement. Alternatively, integrating metasurfaces with resonant cavities could enable multiple interactions between pump light and metasurfaces (*63*) to further boost the photon pair generation efficiency. Furthermore, the ENZ metasurface may be integrated with a compact laser source for further system miniaturization (*64*). The nonlinearity of ENZ metasurfaces can be controlled through electrical tuning (*65*) or ultrafast all-optical modulation (*66*), which may enable the generation of spatiotemporally programmable pairs of polarization-entangled photons (*67*). Furthermore, by engineering the orientation and geometric parameters of the unit cells, the phase difference between signal and idler photons may be controlled flexibly, thus facilitating the generation of hyperentanglement states (*68*).

## MATERIALS AND METHODS

### ENZ metasurface fabrication

A commercially available 23-nm-thick ITO thin film on a float glass (PGO GmbH) is used as the substrate to support the plasmonic nanostructures. The metasurface with a total area of 200 by 200 μm^2^ is fabricated via a commercial service offered by Tianjin H-Chip Technology Group. The SRR pattern is defined using an electron beam lithography system (JEOL JBX-6300FS). A 40-nm-thick gold layer is deposited via electron beam evaporation, followed by a lift-off process. A 5-nm-thick titanium layer is used as an adhesion layer.

### Photon pair generation measurements

A CW laser operating at a wavelength of 775 nm (Toptica DL pro780) is used as the pump light, which is focused onto the metasurface through a high-NA lens (Thorlabs A240TM, *f* = 8 mm, NA = 0.5). The polarization angle of the pump light can be tuned using an HWP (Thorlabs WPHSM05-780). The photon pairs generated from the ENZ metasurface are collected and collimated using an identical lens. Two long-pass filters (Semrock LP1064 and LP1319) and a band-pass filter with a central wavelength of 1550 nm and a bandwidth of 50 nm (Edmund Optics BP1550) are used to block the pump light. The photon pairs are then coupled into the single-mode fiber through a fiber collimator (Thorlabs CFC11A-C, *f* = 11 mm). A linear polarizer is set before the collimator to characterize the polarization state of the photon pairs. After passing through a 50:50 beam splitter, the photon pairs are detected by two independent SNSPDs. To confirm that the pump light is fully blocked by the filters, we remove the ENZ metasurface prior to the measurement, and no signal is detected by the single-photon detectors. Last, we could obtain the coincidence histogram through the TDC. Because the SNSPDs are sensitive to a specific linear polarization state, we first calibrate the setup using a laser with a wavelength of 1550 nm, with its polarization adjusted by an HWP (Thorlabs WPHSM05-1550). Two fiber polarization controllers are used to maximize the photon detection efficiencies of the SNSPDs. In the quantum state tomography measurement, a tilted broadband short-pass filter (Edmund Optics SP1600) is used as a dichroic mirror with a cutoff wavelength of 1550 nm to separate the signal and idler photons deterministically. To mitigate the impact of the incident angle error of the dichroic mirror on the measurements, we remove the band-pass filter in this section.

### SHG measurements

A Ti:sapphire laser is used to pump an optical parametric oscillator (Coherent Chameleon OPO) for generating femtosecond laser pulses with a wavelength of 1550 nm, a pulse duration of 200 fs, and a repetition rate of 80 MHz. A high-NA lens (Thorlabs A240TM, *f* = 8 mm, NA = 0.5) is used to focus the laser onto the ENZ metasurface, resulting in a spot diameter with an FWHM of ~5 μm. The generated second-harmonic wave is collected by a 100× objective (Olympus LMPlanFLN, NA = 0.8) and detected by a visible-range CMOS (complementary metal-oxide semiconductor) camera (Andor Zyla sCMOS). The pump beam is filtered using two short-pass filters (Thorlabs FESH0900). The pump power is set to 0.5 mW. An HWP is used to control the pump polarization state. A linear polarizer is used for characterizing the polarization state of the generated second-harmonic signal.

## Supplementary Materials

### This PDF file includes:

Supplementary Text S1 to S9 Figs. S1 to S7 Tables S1 to S3 References
