# Supplementary material for: Polarization-entangled Bell state generation from an epsilon-near-zero metasurface
Source: Sci Adv. 2025 Feb 21;11(8):eads3576. doi: 10.1126/sciadv.ads3576 (PMC11844724; doi:10.1126/sciadv.ads3576)
Supplement: Supplementary file 1 — Supplementary Text S1 to S9 Figs. S1 to S7 Tables S1 to S3 References [file sciadv.ads3576_sm.pdf]

Science Advances
AAAS

Supplementary Materials for

Polarization-entangled Bell state generation from an epsilon-near-zero metasurface

Wenhe Jia *et al.*

Corresponding author: Rachel Grange, grangera@ethz.ch; Yuanmu Yang, ymyang@tsinghua.edu.cn

Sci. Adv. **11**, eads3576 (2025)
DOI: 10.1126/sciadv.ads3576

This PDF file includes:

Supplementary Text S1 to S9
Figs. S1 to S7
Tables S1 to S3
References

Supplementary Text

Text S1. Linear characteristics of ITO film.

A commercially available ITO film on a float glass substrate (PGO GmbH) is utilized to construct the strongly coupled system with the plasmonic nanostructures. The dispersion curve of ITO film's permittivity is measured via spectroscopic ellipsometry, as shown in Fig. S1, and it follows the Drude model (*41*):

$$\varepsilon_{\mathrm{ITO}}(\omega) = \varepsilon_{\infty} - \frac{{\omega_p}^2}{\omega^2 + i\gamma\omega}, \quad \textbf{(S1)}$$

where ω is the optical frequency, $\varepsilon_{\infty} = 4.42$ is the permittivity at high frequency, $\omega_{\mathrm{p}} = 2.84\times10^{15}$ rad/s is the plasma frequency, and $\gamma = 2.58\times10^{14}$ rad/s is the damping frequency. The real part of its permittivity crosses zero at the wavelength of 1420 nm.

Text S2. Analysis of the strong coupling effect in the ENZ metasurface.

The coupling between the ENZ material and the plasmonic metasurface can be quantitatively determined by the relative resonance frequency splitting $\Delta\nu/\nu_{\mathrm{ENZ}}$, where ν_{ENZ} is the ENZ frequency of the ITO film (*69, 70*). The simulated transmittance spectra as a function of frequency are shown in Fig. S2. The resonance frequency of the SRR without ITO film is 218 THz, which closely matches ν_{ENZ} of 212 THz. When the SRR is coupled to the ITO film, there is a resonance splitting at 192 THz and 238 THz, which corresponds to a $\Delta\nu/\nu_{\mathrm{ENZ}}$ of 22%, indicating the strong coupling effect between the ITO film and SRRs (*69, 70*).

Text S3. Comparison of various sources of pairs of polarization-entangled photons.

Here we benchmark the performance of various sources of pairs of polarization-entangled photons, as summarized in Table S1. Compared with conventional sources of pairs of polarization-entangled photons, the ENZ metasurface is much more compact and simpler to operate, as it does not require strict temperature control. Since the ENZ metasurface is free from the phase-matching constraints, it allows for broader frequency and angular bandwidths. Compared to other recently reported nanoscale sources of pairs of polarization-entangled photons, the ENZ metasurface is the thinnest.

At present, a major challenge of nanoscale photon pair sources is that their absolute photo pair generation efficiency still lags behind that of bulk nonlinear crystals. Despite it being a nascent research field, as is discussed in the main text, one could utilize resonances with higher quality factors such as quasi-bound states in the continuum resonances (*61*) or surface lattice resonances (*62*) to achieve greater field enhancement. Alternatively, integrating metasurfaces with resonant cavities could enable multiple interactions between pump light and metasurfaces (*63*), to further boost the photon pair generation efficiency. Note that in our recent work, we have experimentally demonstrated nearly 1% second-harmonic generation efficiency using the metasurface platform (*71*), despite with a high pump fluence, which gave us some confidence of a steady increase of photon pair generation efficiency based on the metasurface platform as the research field evolve. Furthermore, we would like to re-emphasize that the metasurface platform is distinctive from natural crystals in that it has a highly tailorable anisotropic nonlinear susceptibility tensor, thus may have the potential to inspire a multitude of new directions, including but not limited to the generation of spatiotemporally programmable pairs of polarization-entangled photons and the realization of hyper-entanglement states.

Text S4. Spectral analysis of pairs of entangled photons generated from the ENZ metasurface.
To further explore the spectral characteristics of the generated photon pairs, we perform additional measurements on the coincidence count rate over different spectral bandwidths. Specifically, using a 775 nm laser to pump the ENZ metasurface, the coincidence counts are measured with and without a bandpass filter using the quantum state tomography setup, where the detection ranges are 1525-1575 nm (with the bandpass filter) and 1486-1620 nm (determined by the single-mode fiber's cutoff wavelength), respectively.

Photon pair generation is observed in both cases. As shown in Table S2, when the detection bandwidth increases from 50 nm to 134 nm, the pump power required to achieve comparable detection efficiency decreases from 9.7 mW to 3.5 mW. The calculated detection efficiencies are 0.011 Hz/mW and 0.027 Hz/mW, with the spectrum-normalized efficiencies being 2.2×10^{-4} Hz/(mW·nm) and 2×10^{-4} Hz/(mW·nm), respectively, indicating that the photon pair generation rate remains approximately constant over a 134 nm range. The emission bandwidth of the ENZ metasurface is considerably broader than that of nonlinear bulk crystals, benefiting from the lack of phase-matching constraints (*24*, *27*).

Text S5. $g^{(2)}(0)$ characterization of the generated photon pairs.
To confirm the non-classical nature of the photon pairs generated from the ENZ metasurface, we calculate the second-order correlation function at zero time delay $g^{(2)}(0)$ following the equation (*24*):

$$g^{(2)}(0) = \frac{R_c}{R_s R_i T_c}, \quad \textbf{(S2)}$$

where R_c is the rate of coincidence counts, R_s and R_i are the rates of signal and idler photon counts, respectively, and T_c is the time resolution of the coincidence histogram. As R_c, R_s, and R_i exhibit linear dependence on the pump power, $g^{(2)}(0)$ is inversely proportional to the pump power. Our measurements align with the theoretical power dependence, as depicted in Fig. S3.

Text S6. SPDC measurement for ITO film.
To further confirm that the photon pair generation is enhanced by the resonance and the field amplification induced by the ENZ effect, we compare the coincidence histograms from the ENZ metasurface and the bare ITO film, as shown in Fig. S4. The bare ITO film is pumped with 10 mW at normal incidence with an integration time of one hour, and no obvious peak is observed at zero time delay, indicating that no photon pairs are generated. Since the ENZ effect of the ITO film cannot be excited under normal incidence (*48*), there is no field enhancement within the ITO film. As a result, the photon pairs cannot be effectively generated, which is consistent with the experimental results.

Text S7. Theoretical SHG polarization dependence of the ENZ metasurface.
The ENZ metasurface uses the anisotropic feature of a SRR structure to engineer its effective $\chi^{(2)}$ tensor. When the SRRs are pumped with y-polarized light, the nonlinear polarizations generated within each arm are opposite, leading to destructive interference of SHG signal in the far field, as illustrated in Fig. S5A. In contrast, under x-polarized excitation, the central symmetry of the SRR is broken, and the nonlinear polarizations generated in the two arms have the same polarity. The constructive interference of the nonlinear polarizations in the far field results in a strong y-polarized SHG (*57, 58*), as shown in Fig. S5B.

Based on the above analysis and the Kleinman symmetry, the $\chi^{(2)}$ tensor for the system is constructed as follows: $\chi^{(2)}_{xxx} = 0$, $\chi^{(2)}_{yyy} = 0$, $\chi^{(2)}_{xyy} = \chi^{(2)}_{yyx} = \chi^{(2)}_{yxy} = 0$, and $\chi^{(2)}_{yxx} = \chi^{(2)}_{xxy} = \chi^{(2)}_{xyx} \neq 0$. With this description, we analyze the polarization state of the second-harmonic wave under different pump configurations. The *x*- and *y*-component of the generated SHG are calculated as,

$$E_x^{2\omega} = \chi^{(2)}_{xxy} E_x^{\omega} E_y^{\omega} + \chi^{(2)}_{xyx} E_y^{\omega} E_x^{\omega}, \quad \textbf{(S3)}$$

$$E_y^{2\omega} = \chi^{(2)}_{yxx} E_x^{\omega} E_x^{\omega}, \quad \textbf{(S4)}$$

where $E_x^{\omega} = E_0^{\omega} cos\alpha$ and $E_y^{\omega} = E_0^{\omega} sin\alpha$ are the *x*- and *y*-component of the pump light, respectively, α is the polarization angle of the pump light with respect to the *x*-axis, and E_0 is the amplitude of the pump light.

The electric field component with an angle θ to the *x*-axis of the second-harmonic wave is calculated as,

$$E_\theta^{2\omega} = E_x^{2\omega} cos\theta + E_y^{2\omega} sin\theta. \quad \textbf{(S5)}$$

To compare the theoretical calculations with the measurements, we calculate the SHG intensity as a function of the pump and detection angles following $I_\theta^{2\omega} \propto |E_\theta^{2\omega}|^2$, as shown in Fig. S6. The consistency between theoretical results and measurements (Fig. 4B in the main text) confirms that the nonlinear response of the ENZ metasurface adheres to the derived effective second-order nonlinear susceptibility tensor.

Text S8. Theoretical SPDC polarization dependence of the ENZ metasurface.

According to the derived effective second-order nonlinear susceptibility tensor of the ENZ metasurface, when pumped with linearly polarized light at an angle α to the *x*-axis, the polarization state of the generated photon pair can be expressed as (*27*):

$$|\psi_\alpha\rangle = \frac{1}{\sqrt{1 + cos^2\alpha}} (sin\alpha|HH\rangle + cos\alpha|HV\rangle + cos\alpha|VH\rangle). \quad \textbf{(S6)}$$

When a polarizer is used to measure the polarization state of photons, with its transmission axis at an angle θ with the *x*-axis, its operator can be written in the following form:

$$P(\theta) = |\psi_\theta\rangle\langle\psi_\theta|, \quad \textbf{(S7)}$$

where $|\psi_\theta\rangle = cos\theta|H\rangle + sin\theta|V\rangle$. If the polarizer is used to simultaneously detect both signal and idler photons, its operator can be expressed as:

$$P_{s,i}(\theta) = P(\theta) \otimes P(\theta). \quad \textbf{(S8)}$$

Finally, we can obtain the normalized coincidence counts as a function of the pump and detection angles following:

$$R_{s,i}(\alpha, \theta) = \langle\psi_\alpha|P_{s,i}(\theta)|\psi_\alpha\rangle. \quad \textbf{(S9)}$$

The calculated normalized coincidence counts are shown in Fig. S7. The calculated results for pump polarization angles of ±90° in Fig. S7 correspond to the experimental results presented in Fig. 4C in the main text.

Text S9. Quantum state tomography and fidelity analysis of photon pairs.

To completely determine the polarization states of the generated photon pairs, we choose 16 sets of basic polarization states for the measurements, as summarized in Table S3. Based on the operation matrices $\widehat{M}_i$ provided by James *et al.* (*32*), we reconstruct the density matrix for the polarization state of the photon pairs following (*32*):

$$\rho = \frac{\sum_{i=1}^{16} \widehat{M}_i n_i}{\sum_{i=1}^{4} n_i}, \qquad \textbf{(S10)}$$

where n_i represents the coincidence counts obtained from the *i*-th measurement. For this calculation, we use the open-source code provided by the Kwiat Quantum Information Group. The fidelity of the generated photon pairs is calculated following the equation (*27*):

$$F = \left(tr\left(\sqrt{\rho^{1/2} \tilde{\rho} \rho^{1/2}} \right) \right)^2, \qquad \textbf{(S11)}$$

where ρ and $\tilde{\rho}$ are the reconstructed and theoretical density matrix, respectively.

To calculate the fidelity of two pure states $|\psi_1\rangle$ and $|\psi_2\rangle$, Eq. S11 can be simplified to (*76*):

$$F = |\langle \psi_1 | \psi_2 \rangle|^2. \qquad \textbf{(S12)}$$

For $|\psi_\alpha\rangle$ generated from the ENZ metasurface and the Bell state $|\psi_{Bell}\rangle = \frac{1}{\sqrt{2}}(|HV\rangle + |VH\rangle)$, the fidelity is:

$$F = |\langle \psi_\alpha | \psi_{Bell} \rangle|^2 = \frac{2cos^2\alpha}{1 + cos^2\alpha}. \qquad \textbf{(S13)}$$

Supplementary Figures

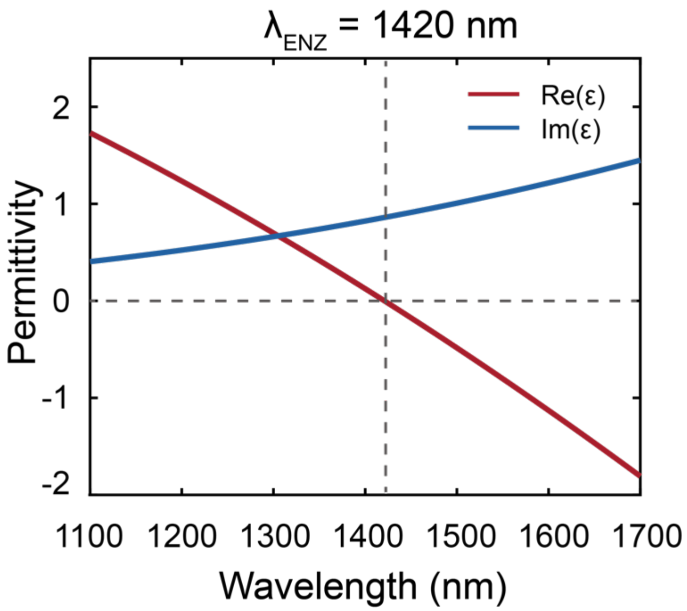

Fig. S1. Real (red) and imaginary (blue) parts of the permittivity of the ITO thin film. The ENZ wavelength of the ITO thin film is measured to be located at 1420 nm, marked with a grey dashed line.

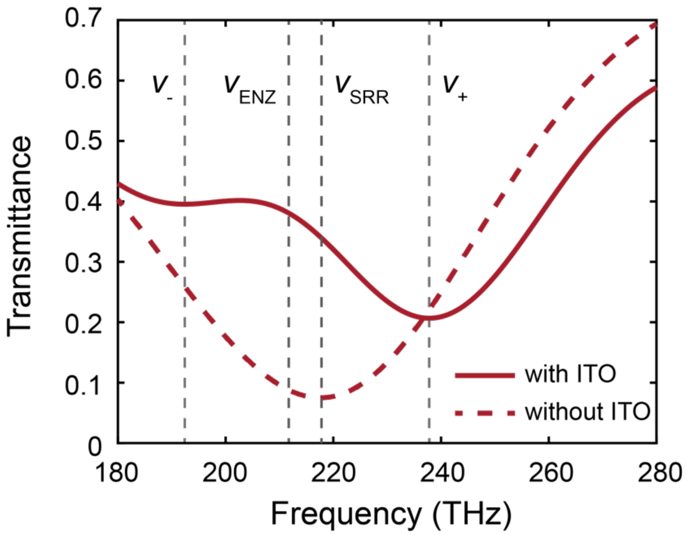

Fig. S2. Simulated linear transmittance spectra of the ENZ metasurface (solid red line) and the SRR without ITO (dashed red line) under *x*-polarized incident light. The grey dashed lines indicate the resonant frequencies of ENZ metasurface (ν_+ and ν_-), SRR without ITO (ν_{SRR}), and the ENZ frequency of ITO film (ν_{ENZ}), respectively.

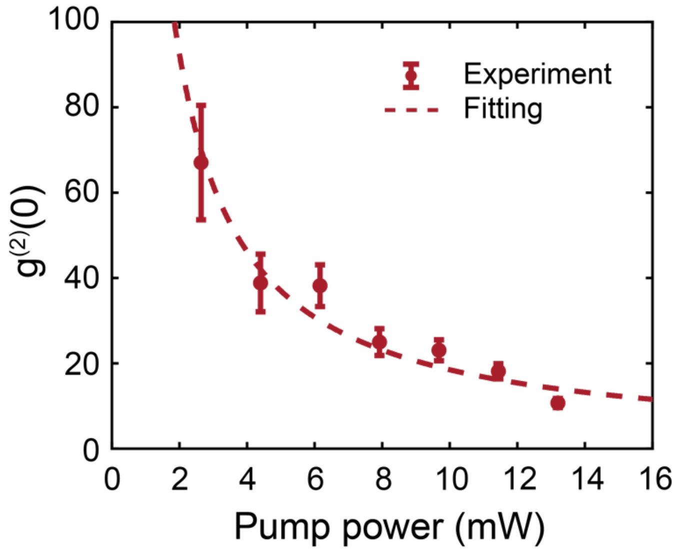

Fig. S3. Measured second-order correlation function at zero time delay $g^{(2)}(0)$ (dots) and the corresponding fitted curve $g^{(2)}(0) \propto \frac{1}{P}$ (line) as a function of the pump power P, indicating an inversely proportional relationship.

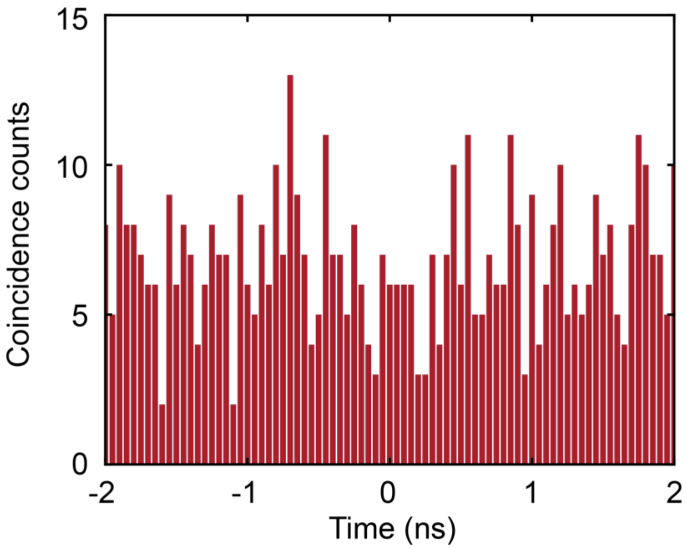

Fig. S4. Measured coincidence histogram from a bare ITO film with a pump power of 10 mW and an integration time of one hour.

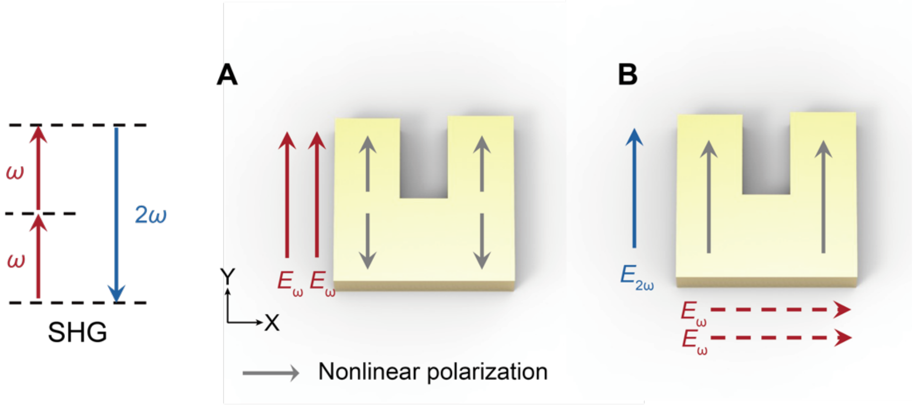

Fig. S5. Analysis of $\chi^{(2)}$ tensor of the SRR. **(A)-(B)** Schematic illustration of the polarization characteristics of the SHG process under *y*- **(A)** and *x*-polarized **(B)** excitation.

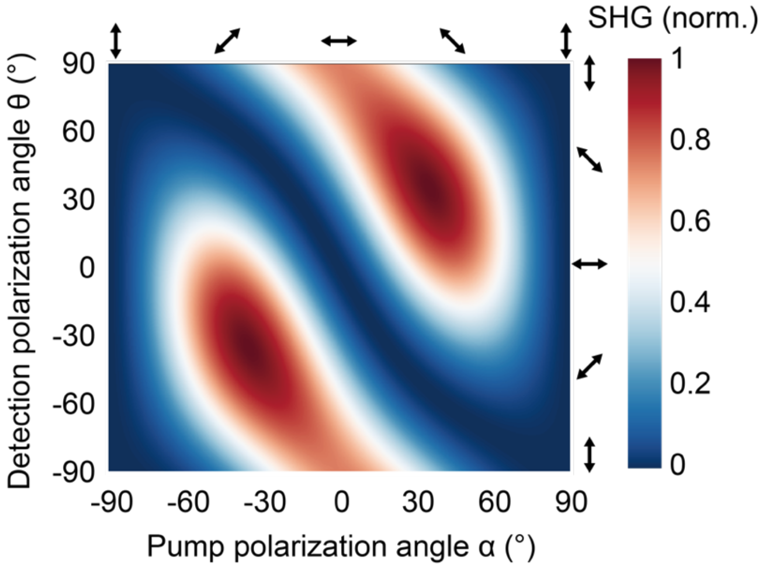

Fig. S6. Calculated SHG intensity from the ENZ metasurface as a function of the pump and the detection polarization angles based on the derived effective second-order nonlinear susceptibility tensor. Both the pump and detection polarization states are linearly polarized, as indicated by the arrows.

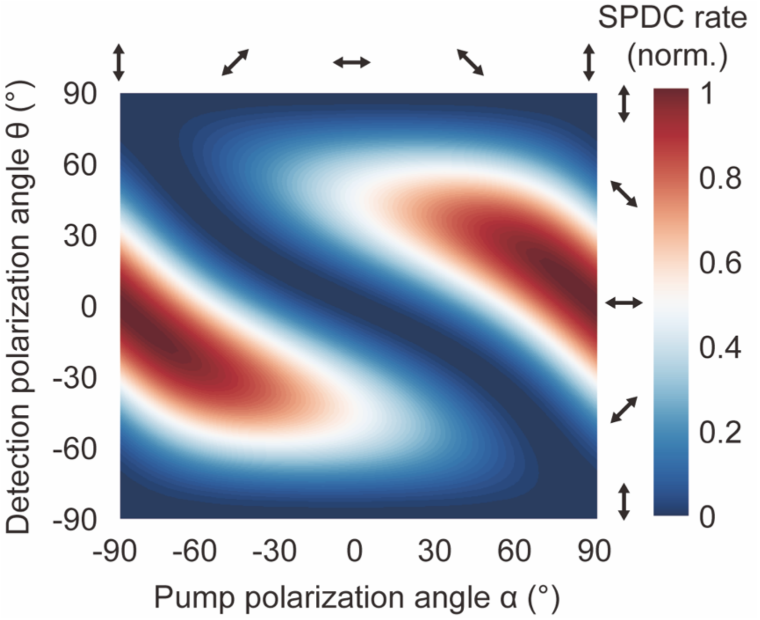

Fig. S7. Calculated SPDC rates from the ENZ metasurface as a function of the pump and the detection polarization angles based on the derived effective second-order nonlinear susceptibility tensor. Both the pump and detection polarization states are linearly polarized, as indicated by the arrows.

Supplementary Tables

Platform	Thickness	Efficiency (Hz/mW)	Phase-matching requirement	Temperature controller
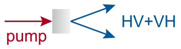<br>BBO (*72*)	2 mm	2.2×10^{2}	Yes	Not needed
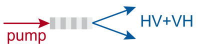<br>PPKTP (*73*)	10 mm	8.2×10^{2}	Yes	Needed
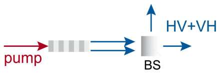<br>PPKTP + post-selective element (*74*)	10 mm	3.0×10^{2}	Yes	Needed
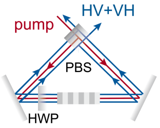<br>PPKTP + interferometer (*17*)	10 mm	5.0×10^{3}	Yes	Needed
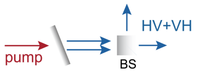<br>GaP film (*75*)	400 nm	8.3×10^{-3}	No	Not needed
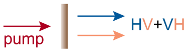<br>3R-stack MoS_2 (*27*)	285 nm	5.7×10^{-3}	No	Not needed
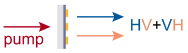<br>ENZ metasurface (this work)	68 nm	5.4×10^{-3}	No	Not needed

Table S1. Comparison of various sources of pairs of polarization-entangled photons.

	**Detection range (nm)**	**Pump power (mW)**	**Coincidence count rate (Hz)**	**Detection efficiency (Hz/mW)**
With bandpass filter	1525-1575	9.7	0.103	0.011
Without bandpass filter	1486-1620	3.5	0.095	0.027

Table S2. Comparison of measurement efficiency with different detection ranges.

Number	Polarization mode 1	Polarization mode 2	QWP 1 (°)	HWP 1 (°)	QWP 2 (°)	HWP 2 (°)
1	$\|H\rangle$	$\|H\rangle$	0	45	0	45
2	$\|H\rangle$	$\|V\rangle$	0	45	0	0
3	$\|V\rangle$	$\|V\rangle$	0	0	0	0
4	$\|V\rangle$	$\|H\rangle$	0	0	0	45
5	$\|R\rangle$	$\|H\rangle$	0	22.5	0	45
6	$\|R\rangle$	$\|V\rangle$	0	22.5	0	0
7	$\|D\rangle$	$\|V\rangle$	45	22.5	0	0
8	$\|D\rangle$	$\|H\rangle$	45	22.5	0	45
9	$\|D\rangle$	$\|R\rangle$	45	22.5	0	22.5
10	$\|D\rangle$	$\|D\rangle$	45	22.5	45	22.5
11	$\|R\rangle$	$\|D\rangle$	0	22.5	45	22.5
12	$\|H\rangle$	$\|D\rangle$	0	45	45	22.5
13	$\|V\rangle$	$\|D\rangle$	0	0	45	22.5
14	$\|V\rangle$	$\|L\rangle$	0	0	90	22.5
15	$\|H\rangle$	$\|L\rangle$	0	45	90	22.5
16	$\|R\rangle$	$\|L\rangle$	0	22.5	90	22.5

Table S3. Basic polarization states and operating angles of quarter-wave plates (QWP) and half-wave plates (HWP) for the quantum state tomography measurements.

REFERENCES AND NOTES

1. A. K. Ekert, Quantum cryptography based on Bell's theorem. *Phys. Rev. Lett.* **67**, 661–663 (1991).

2. V. Scarani, H. Bechmann-Pasquinucci, N. J. Cerf, M. Dusek, N. Lütkenhaus, M. Peev, The security of practical quantum key distribution. *Rev. Mod. Phys.* **81**, 1301–1350 (2009).

3. J. L. O'Brien, A. Furusawa, J. Vučković, Photonic quantum technologies. *Nat. Photonics* **3**, 687–695 (2009).

4. T. C. Ralph, A. Gilchrist, G. J. Milburn, W. J. Munro, S. Glancy, Quantum computation with optical coherent states. *Phys. Rev. A* **68**, 042319 (2003).

5. J. L. O'Brien, Optical quantum computing. *Science* **318**, 1567–1570 (2007).

6. P. Kok, W. J. Munro, K. Nemoto, T. C. Ralph, J. P. Dowling, G. J. Milburn, Linear optical quantum computing with photonic qubits. *Rev. Mod. Phys.* **79**, 135–174 (2007).

7. T. B. Pittman, Y. H. Shih, D. V. Strekalov, A. V. Sergienko, Optical imaging by means of two-photon quantum entanglement. *Phys. Rev. A* **52**, R3429–R3432 (1995).

8. G. B. Lemos, V. Borish, G. D. Cole, S. Ramelow, R. Lapkiewicz, A. Zeilinger, Quantum imaging with undetected photons. *Nature* **512**, 409–412 (2014).

9. C. L. Degen, F. Reinhard, P. Cappellaro, Quantum sensing. *Rev. Mod. Phys.* **89**, 035002 (2017).

10. S. Pirandola, B. R. Bardhan, T. Gehring, C. Weedbrook, S. Lloyd, Advances in photonic quantum sensing. *Nat. Photonics* **12**, 724–733 (2018).

11. X. Guo, C.-L. Zou, C. Schuck, H. Jung, R. Cheng, H. X. Tang, Parametric down-conversion photon-pair source on a nanophotonic chip. *Light Sci. Appl.* **6**, e16249 (2017).

12. S. E. Harris, M. K. Oshman, R. L. Byer, Observation of tunable optical parametric fluorescence. *Phys. Rev. Lett.* **18**, 732–734 (1967).

13. J. W. Silverstone, R. Santagati, D. Bonneau, M. J. Strain, M. Sorel, J. L. O'Brien, M. G. Thompson, Qubit entanglement between ring-resonator photon-pair sources on a silicon chip. *Nat. Commun.* **6**, 7948 (2015).

14. X. Lu, Q. Li, D. A. Westly, G. Moille, A. Singh, V. Anant, K. Srinivasan, Chip-integrated visible–telecom entangled photon pair source for quantum communication. *Nat. Phys.* **15**, 373–381 (2019).

15. A. Anwar, C. Perumangatt, F. Steinlechner, T. Jennewein, A. Ling, Entangled photon-pair sources based on three-wave mixing in bulk crystals. *Rev. Sci. Instrum.* **92**, 041101 (2021).

16. F. Bouchard, A. Sit, Y. Zhang, R. Fickler, F. M. Miatto, Y. Yao, F. Sciarrino, E. Karimi, Two-photon interference: The Hong-Ou-Mandel effect. *Rep. Prog. Phys.* **84**, 012402 (2021).

17. T. Kim, M. Fiorentino, F. N. C. Wong, Phase-stable source of polarization-entangled photons using a polarization Sagnac interferometer. *Phys. Rev. A* **73**, 012316 (2006).

18. G. Li, S. Zhang, T. Zentgraf, Nonlinear photonic metasurfaces. *Nat. Rev. Mater.* **2**, 17010 (2017).

19. A. Krasnok, M. Tymchenko, A. Alu, Nonlinear metasurfaces: A paradigm shift in nonlinear optics. *Mater. Today* **21**, 8–21 (2018).

20. Y. Zhao, Y. Yang, H.-B. Sun, Nonlinear meta-optics towards applications. *PhotoniX* **2**, 3 (2021).

21. A. Di Francescantonio, A. Zilli, D. Rocco, V. Vinel, L. Coudrat, F. Conti, P. Biagioni, L. Duò, A. Lemaître, C. De Angelis, G. Leo, M. Finazzi, M. Celebrano, All-optical free-space routing of upconverted light by metasurfaces via nonlinear interferometry. *Nat. Nanotechnol.* **19**, 298–305 (2023).

22. A. S. Solntsev, G. S. Agarwal, Y. S. Kivshar, Metasurfaces for quantum photonics. *Nat. Photonics* **15**, 327–336 (2021).

23. J. Ma, J. Zhang, J. Horder, A. A. Sukhorukov, M. Toth, D. N. Neshev, I. Aharonovich, Engineering quantum light sources with flat optics. *Adv. Mater.* **36**, e2313589 (2024).

24. C. Okoth, A. Cavanna, T. Santiago-Cruz, M. V. Chekhova, Microscale generation of entangled photons without momentum conservation. *Phys. Rev. Lett.* **123**, 263602 (2019).

25. T. Santiago-Cruz, A. Fedotova, V. Sultanov, M. A. Weissflog, D. Arslan, M. Younesi, T. Pertsch, I. Staude, F. Setzpfandt, M. Chekhova, Photon pairs from resonant metasurfaces. *Nano Lett.* **21**, 4423–4429 (2021).

26. Q. Guo, X. Z. Qi, L. Zhang, M. Gao, S. Hu, W. Zhou, W. Zang, X. Zhao, J. Wang, B. Yan, M. Xu, Y. K. Wu, G. Eda, Z. Xiao, S. A. Yang, H. Gou, Y. P. Feng, G. C. Guo, W. Zhou, X. F. Ren, C. W. Qiu, S. J. Pennycook, A. T. S. Wee, Ultrathin quantum light source with van der Waals $NbOCl_2$ crystal. *Nature* **613**, 53–59 (2023).

27. M. A. Weissflog, A. Fedotova, Y. Tang, E. A. Santos, B. Laudert, S. Shinde, F. Abtahi, M. Afsharnia, I. Perez Perez, S. Ritter, H. Qin, J. Janousek, S. Shradha, I. Staude, S. Saravi, T. Pertsch, F. Setzpfandt, Y. Lu, F. Eilenberger, A tunable transition metal dichalcogenide entangled photon-pair source. *Nat. Commun.* **15**, 7600 (2024).

28. T. Santiago-Cruz, S. D. Gennaro, O. Mitrofanov, S. Addamane, J. Reno, I. Brener, M. V. Chekhova, Resonant metasurfaces for generating complex quantum states. *Science* **377**, 991–995 (2022).

29. J. Zhang, J. Ma, M. Parry, M. Cai, R. Camacho-Morales, L. Xu, D. N. Neshev, A. A. Sukhorukov, Spatially entangled photon pairs from lithium niobate nonlocal metasurfaces. *Sci. Adv.* **8**, eabq4240 (2022).

30. C. Son, V. Sultanov, T. Santiago-Cruz, A. P. Anthur, H. Z. Zhang, R. Paniagua-Dominguez, L. Krivitsky, A. I. Kuznetsov, M. V. Chekhova, Photon pairs bi-directionally emitted from a resonant metasurface. *Nanoscale* **15**, 2567–2572 (2023).

31. M. A. Weissflog, J. Ma, J. Zhang, T. Fan, S. Lung, T. Pertsch, D. N. Neshev, S. Saravi, F. Setzpfandt, A. A. Sukhorukov, Directionally tunable co-and counterpropagating photon pairs from a nonlinear metasurface. *Nanophotonics* **13**, 3563–3573 (2024).

32. D. F. V. James, P. G. Kwiat, W. J. Munro, A. G. White, Measurement of qubits. *Phys. Rev. A* **64**, 052312 (2001).

33. J. Yin, Y.-H. Li, S.-K. Liao, M. Yang, Y. Cao, L. Zhang, J.-G. Ren, W.-Q. Cai, W.-Y. Liu, S.-L. Li, R. Shu, Y.-M. Huang, L. Deng, L. Li, Q. Zhang, N.-L. Liu, Y.-A. Chen, C.-Y. Lu, X.-B. Wang, F. Xu, J.-Y. Wang, C.-Z. Peng, A. K. Ekert, J.-W. Pan, Entanglement-based secure quantum cryptography over 1,120 kilometres. *Nature* **582**, 501–505 (2020).

34. J. Ma, J. Zhang, Y. Jiang, T. Fan, M. Parry, D. N. Neshev, A. A. Sukhorukov, Polarization engineering of entangled photons from a lithium niobate nonlinear metasurface. *Nano Lett.* **23**, 8091–8098 (2023).

35. M. A. Weissflog, R. Dezert, V. Vinel, C. Gigli, G. Leo, T. Pertsch, F. Setzpfandt, A. Borne, S. Saravi, Nonlinear nanoresonators for Bell state generation. *Appl. Phys. Rev.* **11**, 011403 (2024).

36. M. Kauranen, A. V. Zayats, Nonlinear plasmonics. *Nat. Photonics* **6**, 737–748 (2012).

37. N. Meinzer, W. L. Barnes, I. R. Hooper, Plasmonic meta-atoms and metasurfaces. *Nat. Photonics* **8**, 889–898 (2014).

38. M. Celebrano, X. Wu, M. Baselli, S. Grossmann, P. Biagioni, A. Locatelli, C. De Angelis, G. Cerullo, R. Osellame, B. Hecht, L. Duo, F. Ciccacci, M. Finazzi, Mode matching in multiresonant plasmonic nanoantennas for enhanced second harmonic generation. *Nat. Nanotechnol.* **10**, 412–417 (2015).

39. Y. Ming, W. Zhang, J. Tang, Y. Liu, Z. Xia, Y. Liu, Y.-Q. Lu, Photonic entanglement based on nonlinear metamaterials. *Laser Photonics Rev.* **14**, 1900146 (2020).

40. B. Y. Jin, D. Mishra, C. Argyropoulos, Efficient single-photon pair generation by spontaneous parametric down-conversion in nonlinear plasmonic metasurfaces. *Nanoscale* **13**, 19903–19914 (2021).

41. M. Z. Alam, I. De Leon, R. W. Boyd, Large optical nonlinearity of indium tin oxide in its epsilon-near-zero region. *Science* **352**, 795–797 (2016).

42. I. Liberal, N. Engheta, Near-zero refractive index photonics. *Nat. Photonics* **11**, 149–158 (2017).

43. O. Reshef, I. De Leon, M. Z. Alam, R. W. Boyd, Nonlinear optical effects in epsilon-near-zero media. *Nat. Rev. Mater.* **4**, 535–551 (2019).

44. N. Kinsey, C. DeVault, A. Boltasseva, V. M. Shalaev, Near-zero-index materials for photonics. *Nat. Rev. Mater.* **4**, 742–760 (2019).

45. J. B. Khurgin, M. Clerici, V. Bruno, L. Caspani, C. DeVault, J. Kim, A. Shaltout, A. Boltasseva, V. M. Shalaev, M. Ferrera, D. Faccio, N. Kinsey, Adiabatic frequency shifting in epsilon-near-zero materials: The role of group velocity. *Optica* **7**, 226–231 (2020).

46. A. Capretti, Y. Wang, N. Engheta, L. Dal Negro, Comparative study of second-harmonic generation from epsilon-near-zero indium tin oxide and titanium nitride nanolayers excited in the near-infrared spectral range. *ACS Photonics* **2**, 1584–1591 (2015).

47. Y. Yang, J. Lu, A. Manjavacas, T. S. Luk, H. Liu, K. Kelley, J.-P. Maria, E. L. Runnerstrom, M. B. Sinclair, S. Ghimire, I. Brener, High-harmonic generation from an epsilon-near-zero material. *Nat. Phys.* **15**, 1022–1026 (2019).

48. W. Jia, M. Liu, Y. Lu, X. Feng, Q. Wang, X. Zhang, Y. Ni, F. Hu, M. Gong, X. Xu, Y. Huang, W. Zhang, Y. Yang, J. Han, Broadband terahertz wave generation from an epsilon-near-zero material. *Light Sci. Appl.* **10**, 11 (2021).

49. M. Z. Alam, S. A. Schulz, J. Upham, I. De Leon, R. W. Boyd, Large optical nonlinearity of nanoantennas coupled to an epsilon-near-zero material. *Nat. Photonics* **12**, 79–83 (2018).

50. J. Deng, Y. Tang, S. Chen, K. Li, A. V. Zayats, G. Li, Giant enhancement of second-order nonlinearity of epsilon-near-zero medium by a plasmonic metasurface. *Nano Lett.* **20**, 5421–5427 (2020).

51. W. Jia, C. Gao, Y. Zhao, L. Li, S. Wen, S. Wang, C. Bao, C. Jiang, C. Yang, Y. Yang, Intracavity spatiotemporal metasurfaces. *Adv. Photonics* **5**, 026002 (2023).

52. F. X. Wang, F. J. Rodríguez, W. M. Albers, R. Ahorinta, J. E. Sipe, M. Kauranen, Surface and bulk contributions to the second-order nonlinear optical response of a gold film. *Phys. Rev. B* **80**, 233402 (2009).

53. L. Rodríguez-Suné, M. Scalora, A. S. Johnson, C. Cojocaru, N. Akozbek, Z. J. Coppens, D. Perez-Salinas, S. Wall, J. Trull, Study of second and third harmonic generation from an indium tin oxide nanolayer: Influence of nonlocal effects and hot electrons. *APL Photonics* **5**, 010801 (2020).

54. G. Saerens, T. Dursap, I. Hesner, N. M. H. Duong, A. S. Solntsev, A. Morandi, A. Maeder, A. Karvounis, P. Regreny, R. J. Chapman, A. Danescu, N. Chauvin, J. Penuelas, R. Grange, Background-free near-infrared biphoton emission from single GaAs nanowires. *Nano Lett.* **23**, 3245–3250 (2023).

55. N. M. Hanh Duong, G. Saerens, F. Timpu, M. T. Buscaglia, V. Buscaglia, A. Morandi, J. S. Müller, A. Maeder, F. Kaufmann, A. S. Solntsev, R. Grange, Spontaneous parametric down-conversion in bottom-up grown lithium niobate microcubes. *Opt. Mater. Express* **12**, 3696–3704 (2022).

56. O. A. Ivanova, T. S. Iskhakov, A. N. Penin, M. V. Chekhova, Multiphoton correlations in parametric down-conversion and their measurement in the pulsed regime. *Quantum Electron.* **36**, 951–956 (2006).

57. M. W. Klein, C. Enkrich, M. Wegener, S. Linden, Second-harmonic generation from magnetic metamaterials. *Science* **313**, 502–504 (2006).

58. C. Ciracì, E. Poutrina, M. Scalora, D. R. Smith, Origin of second-harmonic generation enhancement in optical split-ring resonators. *Phys. Rev. B* **85**, 201403 (2012).

59. R. W. Boyd, *Nonlinear Optics, Fourth Edition* (Academic Press, 2020).

60. G. Marino, A. S. Solntsev, L. Xu, V. F. Gili, L. Carletti, A. N. Poddubny, M. Rahmani, D. A. Smirnova, H. Chen, A. Lemaître, G. Zhang, A. V. Zayats, C. De Angelis, G. Leo, A. A. Sukhorukov, D. N. Neshev, Spontaneous photon-pair generation from a dielectric nanoantenna. *Optica* **6**, 1416–1422 (2019).

61. Y. Liang, K. Koshelev, F. Zhang, H. Lin, S. Lin, J. Wu, B. Jia, Y. Kivshar, Bound states in the continuum in anisotropic plasmonic metasurfaces. *Nano Lett.* **20**, 6351–6356 (2020).

62. M. S. Bin-Alam, O. Reshef, Y. Mamchur, M. Z. Alam, G. Carlow, J. Upham, B. T. Sullivan, J. M. Menard, M. J. Huttunen, R. W. Boyd, K. Dolgaleva, Ultra-high-Q resonances in plasmonic metasurfaces. *Nat. Commun.* **12**, 974 (2021).

63. M. Mekhael, T. Stolt, A. Vesala, H. Rekola, T. K. Hakala, R. Fickler, M. J. Huttunen, Phase-matched second-harmonic generation from metasurfaces inside multipass cells. *ACS Photonics* **11**, 682–687 (2024).

64. Y. Y. Xie, P. N. Ni, Q. H. Wang, Q. Kan, G. Briere, P. P. Chen, Z. Z. Zhao, A. Delga, H. R. Ren, H. D. Chen, C. Xu, P. Genevet, Metasurface-integrated vertical cavity surface-emitting lasers for programmable directional lasing emissions. *Nat. Nanotechnol.* **15**, 125–130 (2020).

65. Y.-W. Huang, H. W. H. Lee, R. Sokhoyan, R. A. Pala, K. Thyagarajan, S. Han, D. P. Tsai, H. A. Atwater, Gate-tunable conducting oxide metasurfaces. *Nano Lett.* **16**, 5319–5325 (2016).

66. A. Y. Bykov, J. Deng, G. Li, A. V. Zayats, Time-dependent ultrafast quadratic nonlinearity in an epsilon-near-zero platform. *Nano Lett.* **24**, 3744–3749 (2024).

67. V. Sultanov, A. Kavcic, E. Kokkinakis, N. Sebastian, M. V. Chekhova, M. Humar, Tunable entangled photon-pair generation in a liquid crystal. *Nature* **631**, 294–299 (2024).

68. J. T. Barreiro, N. K. Langford, N. A. Peters, P. G. Kwiat, Generation of hyperentangled photon pairs. *Phys. Rev. Lett.* **95**, 260501 (2005).

69. S. Campione, J. R. Wendt, G. A. Keeler, T. S. Luk, Near-infrared strong coupling between metamaterials and epsilon-near-zero modes in degenerately doped semiconductor nanolayers. *ACS Photonics* **3**, 293–297 (2016).

70. S. Campione, S. Liu, A. Benz, J. F. Klem, M. B. Sinclair, I. Brener, Epsilon-near-zero modes for tailored light-matter interaction. *Phys. Rev. Appl.* **4**, 044011 (2015).

71. Y. Zhao, Z. X. Chen, C. Wang, Y. M. Yang, H. B. Sun, Efficient second- and higher-order harmonic generation from $LiNbO_3$ metasurfaces. *Nanoscale* **15**, 12926–12932 (2023).

72. P. Trojek, C. Schmid, M. Bourennane, H. Weinfurter, C. Kurtsiefer, Compact source of polarization-entangled photon pairs. *Opt. Express* **12**, 276–281 (2004).

73. M. Fiorentino, C. E. Kuklewicz, F. N. C. Wong, Source of polarization entanglement in a single periodically poled $KTiOPO_4$ crystal with overlapping emission cones. *Opt. Express* **13**, 127–135 (2005).

74. C. E. Kuklewicz, M. Fiorentino, G. Messin, F. N. C. Wong, J. H. Shapiro, High-flux source of polarization-entangled photons from a periodically poled $KTiOPO_4$ parametric down-converter. *Phys. Rev. A* **69**, 013807 (2004).

75. V. Sultanov, T. Santiago-Cruz, M. V. Chekhova, Flat-optics generation of broadband photon pairs with tunable polarization entanglement. *Opt. Lett.* **47**, 3872–3875 (2022).

76. J. B. Altepeter, E. R. Jeffrey, P. G. Kwiat, Photonic state tomography. *Adv. At. Mol. Opt. Phys.* **52**, 105–159 (2005).
